# Effect of High Dietary Salt Intake on Macro-Mineral Status of Lactating Camels Raised Under Arid Conditions

**DOI:** 10.3390/vetsci12111026

**Published:** 2025-10-23

**Authors:** Riyadh S. Aljumaah, Moez Ayadi, Abdulkareem M. Matar, Ahmed A. K. Salama, Gerardo Caja, Mohammed A. Alshaikh, Mutassim M. Abdelrahman

**Affiliations:** 1 Department of Animal Production, College of Food and Agriculture Sciences, King Saud University, P.O. Box 2460, Riyadh 11451, Saudi Arabia; 2 Department of Animal Biotechnology, Higher Institute of Biotechnology of Beja, University of Jendouba, Beja 9000, Tunisia; 3 Group of Research in Ruminants (G2R), Department of Animal and Food Sciences, Universitat Autònoma de Barcelona, 08193 Bellaterra, Spain

**Keywords:** blood serum, camel, milk components, macro-minerals and salt (NaCl)

## Abstract

This study examined the effects of high dietary salt intake on the concentrations of major microminerals (K, Na, P, Mg, and Ca) in the serum and milk of lactating dromedary camels (*Camelus dromedarius*). Twelve multiparous camels were assigned in a two-period crossover design to receive either a control diet containing 1.5% NaCl (CON) or a salt-supplemented diet containing 4.5% NaCl (T1). Each period lasted 21 days, including 14 days of adaptation and 7 days of sampling. Blood and milk samples were analyzed using inductively coupled serum–mass spectrometry (ICP-MS). Camels fed the high-salt diet showed lower serum concentrations of Mg and Ca (*p* < 0.05). Milk concentrations of Mg, K, and Ca increased under high-salt intake (*p* < 0.05), whereas Na and P decreased. Correlation analysis revealed negative associations between P intake and milk Ca, as well as between milk Ca and serum Mg. While positive associations were observed between K intake and milk Ca, and between Ca and Mg within milk. Principal component analysis further demonstrated distinct clustering of minerals according to dietary treatment. These results highlight the complex regulation of mineral partitioning between serum and milk in camels exposed to saline diets, underscoring their adaptive capacity to arid environments. The findings provide insights for developing mineral supplementation strategies tailored to camel production systems in regions with high dietary salinity.

## Simple Summary

Camels’ diet is an important factor in producing high-quality dairy products. Milk provides essential minerals and is a major source of bioavailable calcium (Ca) in the diet. Salt (NaCl) supplements are an essential component of animal nutrition. This experiment evaluated the effect of adding two levels of salt (CON = 1.5% of salt and T1 = 4.5% of salt) to the camel diet on serum and milk micromineral concentrations. High dietary salt intake significantly altered mineral dynamics in lactating camels, with increased concentrations of magnesium (Mg) and Ca in blood serum but reduced Mg, potassium (K), and Ca concentrations in camel milk.

## 1. Introduction

Milk is a complex biological fluid that provides both nutritional and immunological protection to neonates [1]. Milk composition includes proteins, lipids, carbohydrates, vitamins, and minerals and varies with species, stage of lactation, and environmental conditions [2]. In camel milk, the mineral fraction typically ranges from 0.6 to 2.5 g/L, constituting an important contribution to human dietary requirements [2]. Minerals are distributed across different milk phases: soluble salts in the aqueous fraction, calcium (Ca) and phosphorus in colloidal micelles, and trace elements in fat globule membranes. While many mineral concentrations remain relatively stable due to homeostatic regulation, others are more sensitive to diet or physiological state [3].

From a nutritional standpoint, milk is the most important dietary source of bioavailable Ca and provides a range of other essential minerals for human health. In camel milk, the total mineral content ranges from 0.58 to 2.48 g/L [3]. Physically and chemically, milk is a multiphase system existing as a true solution, colloidal suspension, and emulsion. Carbohydrates, soluble minerals, and water-soluble vitamins are dissolved in the aqueous phase; proteins and part of the mineral fraction are organized in colloidal structures (mainly casein micelles); and fats and fat-soluble vitamins are dispersed as emulsified globules [1]. The distribution of minerals across the phases of milk production reflects their chemical nature. In the aqueous fraction, salts of Ca, K, Mg, Na, and Fe, and trace elements such as Fe, are present alongside lactose and soluble nitrogenous compounds [4]. In the colloidal phase, casein micelles harbor approximately 20% of the Ca and P, bound to colloidal calcium phosphate and citrate complexes, ensuring structural stability. Fat globule membranes are enriched with phospholipids and trace minerals such as Fe, Cu, Zn, and Mn [5]. Interestingly, mineral concentrations in milk are relatively stable and not strongly affected by dietary fluctuations or seasonal variation. Calcium (Ca), phosphorus (P), and magnesium (Mg) levels are largely homeostatically regulated, as the animal can mobilize bone reserves when necessary. Similarly, sodium (Na), potassium (K), and chloride concentrations remain tightly controlled to maintain osmotic equilibrium between blood and milk [6]. Trace element concentrations, however, can reflect dietary supply: deficiencies in Fe, Zn, or Cu lead to lower milk levels, while supplementation of elements such as Co, Se, I, or Mo increases their concentration. During late lactation, mineral contents of Ca, P, Na, and Cl generally rise [6]. Milk mineral fractions also interact dynamically with the protein matrix, affecting both product quality and nutritional value. Variability in milk composition arises from the interplay of environmental (season and photoperiod), managerial (milking stage), nutritional (feed type, quality, and frequency), and genetic factors (breed, lactation number, and animal health status) [7].

Camels obtain most of the minerals they require from the environment, grazing, water, and particles adhering to their feed [8]. Therefore, effective mineral nutrition management requires supplying minerals in amounts that meet the camel’s physiological needs. This may be assessed by measuring minerals in various tissues of the animal to determine whether intake is adequate [9]. Mineral absorption studies indicate that the reticulorumen is the major site of Ca^2+^ and Mg^2+^ absorption, while P and K absorption occur primarily in the small and large intestines [10]. This factor demonstrates that efficient mineral absorption is directly related to optimal gut function, which can be controlled by diet and stimulation of normal ruminal gut function.

The physiology of camels diverges from that of other ruminants, not only in rumen-reticulum structure but also in adaptive mechanisms that enable survival under extreme conditions. This uniqueness extends to mineral metabolism, influencing absorption, bioavailability, and excretion [11]. Understanding these processes is critical for establishing accurate nutritional requirements for camels, rather than extrapolating from cattle or small ruminants.

Biochemical analysis of blood components helps clinicians establish standardized reference values that facilitate the assessment of animal health status [12]. Metabolites determined from blood as an indicator of dietary energy intake include glucose (GLC), triglycerides (TG), cholesterol (CHL), total protein (TP), and physiological substances such as urea, globulin (GLB), albumin (ALB), and creatinine. Changes in these indicators related to age, disease, nutrition, physiological status, and environmental conditions were reported in camels [13].

One of the major challenges for camels in arid regions is the high salinity of natural forages. Their ability to tolerate and adapt to saline diets affects mineral homeostasis, blood chemistry, and mineral transfer into milk. Within this context, the present study aimed to evaluate the impact of high salt intake on the concentrations of macro-minerals (K, Na, P, Mg, Ca) in both serum and milk of camels raised under arid Saudi Arabian conditions. Additionally, correlations between serum and milk mineral levels were investigated to provide insights into mineral partitioning and transfer dynamics.

## 2. Materials and Methods

The experiment was carried out at Al-Kharj camel farm under the supervision of the Faculty of Agriculture, Animal Production Department, King Saud University in Riyadh. The Governorate of Al-Kharj has a continental climate characterized by very hot summers and dry and cold winters with minimal precipitation. All animal handling procedures were performed in accordance with the guidelines on the care and use of laboratory animals and on the humane treatment of animals with hygienic handling. All animals were tested for mastitis using the California Mastitis Test (CMT) to ensure that they were free from mastitis.

### 2.1. Experimental Design

The experiment was carried out in 2023 between February and April with moderate daytime temperatures during the day and low temperatures at night. All the animals were placed in two pens with six animals in each pen, each with a surface area of 12 × 8 m^2^, each containing a long communal trough for feeding and a water tank with a capacity of about 400 L per day, refilled every morning.

The study involved twelve multiparous lactating camels with body weight estimated (578 ± 24 kg), parities (3.1 ± 0.3), and days in milk (105 ± 22) randomly assigned to two groups of six animals. A two-treatment, two-period crossover design was employed to evaluate the effects of dietary salt levels. The control diet (CON) contained 1.4–1.5% salt, while the high salt-supplemented diet (T1) contained 4.3–4.5% salt, both with 99.7% purity. Each period lasted 21 days, comprising 14 days of adaptation followed by 7 days of measurement. In the first period, one group received CON and the other T1; the treatments were reversed in the second period, thus allowing each animal to serve as its own control [14]. Camels were fed a daily 6.5 kg concentrate pelleted diet (1.5% salt = 9.75 g/day salt, and 4.5% = 29.25 g/day salt) and approximately 3.8 kg alfalfa hay. Concentrated feeding stuff is introduced after morning milking at 6.5 kg per camel, while alfalfa hay is introduced after 2 to 3 h. Camels consumed all the offered feed, with no refusals recorded. The feeding regime was designed to cover the nutrient requirements and ensure that the camels consumed the same amount of salt in each group.

### 2.2. Sample Collection

Camels were milked twice a day (at 9 a.m. and 5 p.m.), and milk yield was recorded at each milking throughout the 7-day measurement phase. Composite milk samples were collected on days 5, 6, and 7 of each period. Blood samples were obtained by jugular venipuncture after morning milking and before feeding on the last day of each period using a vacutainer tube, and serum was separated by centrifugation for 10 min at 3000 rpm and stored at −20 °C until mineral analysis.

### 2.3. Diet Composition

The ingredients, chemical composition, and elements of the experimental diets under investigation were obtained through direct measurement as reported in Table 1 on a dry matter basis.

### 2.4. Sample Preparation for Mineral Analysis

Milk, serum, and feed samples were prepared following the method of [16]. Minerals (Mg, K, Ca, Na, and P) were quantified using inductively coupled serum–mass spectrometry (ICP-MS; Nexion 300D, PerkinElmer, Waltham, MA, USA). Milk (1 mL) and feed (1 g) samples were digested with a mixture of nitric acid (3 mL), deionized water (1 mL), hydrochloric acid (1 mL), and hydrogen peroxide (1 mL) on a hot plate at 122 °C for up to 3 h. After cooling, the digested solutions were diluted to 25 mL with 0.1 N HCl before analysis. Serum samples were analyzed directly. Analytical-grade reagents and Milli-Q water (18 MΩ; Milli-Q Genesis, Billerica, MA, USA) were used throughout. Instrument calibration was performed using ICP-MS standards diluted to 10 mg/L in 2% HNO_3_. All measurements were performed in triplicate, and samples with a deviation exceeding 5% were reanalyzed. The metabolism of the blood, including glucose, total protein, albumin, globulin, triglycerides, and urea, was analyzed by the Química Clínica (Tarragona, Spain) kits by colorimetric means using the RX Monza chemical analyzer (Randox Laboratories Ltd., Crumlin, UK).

### 2.5. Statistical Analysis

Data are expressed as mean ± standard error of the mean (SEM). A repeated-measures analysis of variance (ANOVA) was performed using the PROC-MIXED by SAS (version 9.4; SAS Institute Inc., Cary, NC, USA), with treatment, period, and their interaction included. Because the experimental design was a crossover between two treatment periods, the interaction treatment × period took into account the potential residual effect of the previous treatment period. Residuals were examined for normality, and data were transformed when necessary to meet model assumptions. Statistical significance was declared at *p* < 0.05, and trends were discussed at 0.05 ≤ *p* < 0.10. Pearson’s correlation coefficients were calculated to assess the relationships among mineral concentrations in feed, serum, and milk. Multivariate analysis was performed using principal component analysis (PCA) to identify patterns of association and clustering among minerals across treatments and physiological states. The first two principal components (PC1 and PC2) were retained based on eigenvalues > 1.0 and the proportion of explained variance, and dimensional plots were constructed to visualize mineral groupings.

## 3. Results

The results shown in Table 2 indicate that the high salt content added to the concentrate did not affect milk production, as it was accompanied by a significant increase in water consumption of 5.3% in the T1 group compared to the CON group during two periods, as reported in a previous study [14]. On the other hand, the animals were healthy at the end of the experiment, and there was no loss of weight or appetite.

The effects of dietary salt treatments on serum mineral concentrations of lactating camels during the two milking periods are presented in Table 3. No significant differences were observed in serum K, Na, and P concentrations between treatments (*p* > 0.05), although values tended to be higher in camels receiving the high-salt diet (T1). In contrast, serum Mg and Ca concentrations were significantly reduced (*p* < 0.05) in camels fed the high-salt diet compared with those fed the control diet (CON).

As shown in Table 4, the concentrations of milk minerals in camels subjected to the salt treatments during the two periods varied according to diet. The Mg, K, and Ca concentrations were significantly higher (*p* < 0.05) in the milk of camels fed the high-salt diet (T1). In contrast, the Na concentration tended to decrease under the high-salt treatment (T1; *p* > 0.05). These results were opposite to the trends observed in the serum mineral profile of the same camels. Furthermore, no significant effect of milking period was detected on milk micromineral concentrations under either treatment.

**Figure 1:**
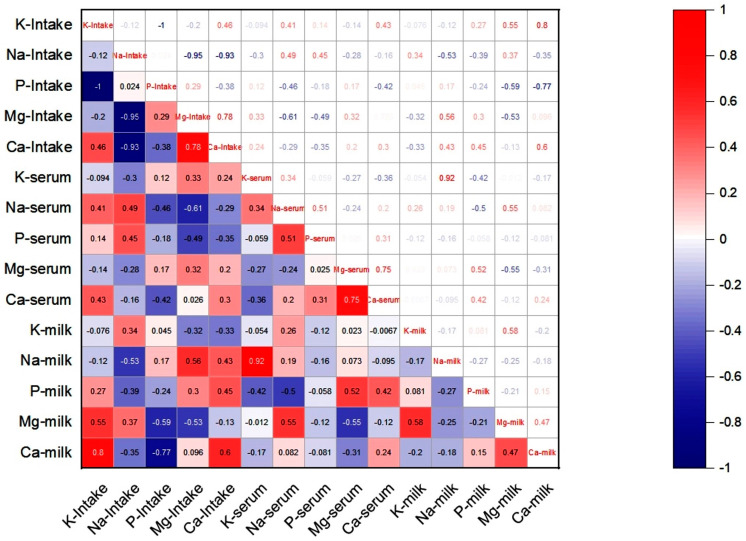
Correlation coefficients (r) among macro mineral content in intake, milk, and blood serum of camels fed with different concentrate levels of salt (1.5 and 4.5%). Correlation is significant at the *p* < 0.05 level.

The correlation coefficients (Figure 1) between mineral contents of feed, serum, and milk in camels supplemented with different levels of dietary salt are presented in Figure 1. Overall, salt (NaCl) and macro mineral intake from feed showed no significant relationship (*p* > 0.05) with serum or milk mineral concentrations. However, several specific associations were observed. A negative correlation was found between P intake and milk Ca content (r = −0.772, *p* = 0.024), while K intake was positively correlated with milk Ca content (r = 0.799, *p* = 0.017). Milk Ca was also positively correlated with milk Mg (r = 0.641, *p* = 0.007). In addition, milk Na was positively correlated with serum K (r = 0.92, *p* = 0.001), whereas serum P was positively correlated with serum Na (r = 0.604, *p* = 0.013).

Serum concentrations of glucose, total protein, globulin, albumin, urea, and triglycerides were unaffected by high salt concentrate, but there was a significant effect of period on urea and triglycerides (*p* < 0.05) in camels consuming diets containing high salt supplementation (Table 5).

**Figure 2:**
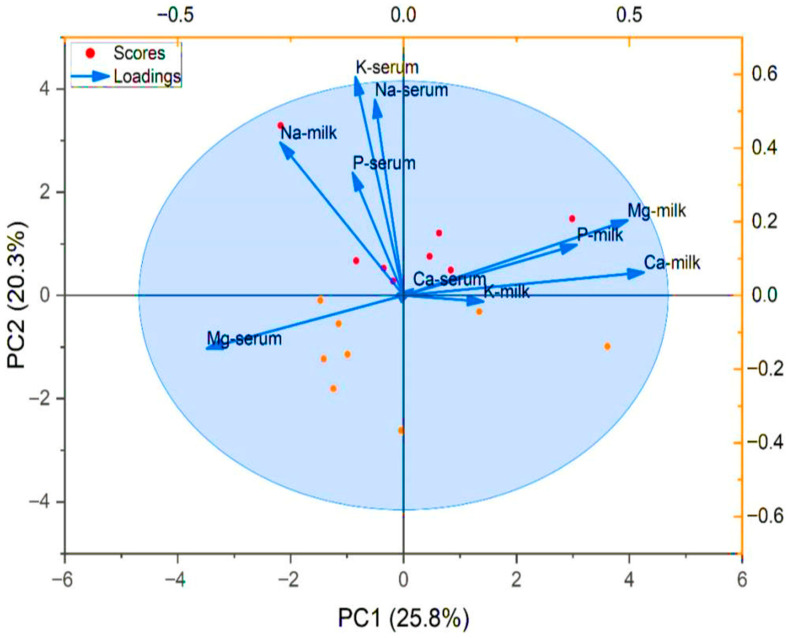
Principal component analysis loading plots for two treatments of components salt 1.5 and 4.5% and associated with macro minerals (K, Na, P, Mg, and Ca) in camel serum and milk.

Multivariate analysis, using principal components (Figure 2), added another dimension by reducing the complexity of mineral variables and highlighting patterns of association. The separation of serum Mg into an independent cluster and the grouping of Na, K, and P between serum and milk indicated specific regulatory pathways under high-salt intake. Together, these analyses confirmed that camel mineral metabolism is not only influenced by dietary salt intake but also governed by intricate interrelationships among macro-minerals, particularly Ca–Mg and Na–K balances.

## 4. Discussion

Minerals are vital for structural development, enzymatic activity, and osmotic regulation in animals. Mineral deficiencies and nutritional imbalances affect production, reproduction, and overall herd health by disrupting the function of biomolecules and tissues, metabolism, and fertility [17]. Minerals account for about 5% of body weight, with more than 26 elements recognized as nutritionally essential and classified as macro-, micro-, or trace elements [18]. In ruminants, mineral imbalances may arise from insufficient dietary intake or antagonistic interactions that reduce absorption and utilization [19]. These risks are particularly pronounced during lactation, when requirements increase while voluntary feed intake may decline [2].

Camel mineral metabolism is less well characterized than that of cattle or sheep, but available evidence indicates that deficiencies of Ca, P, or Mg can compromise productivity, reproduction, and metabolic balance [20,21]. Current supplementation practices are often extrapolated from dairy cattle, despite camels’ unique adaptive physiology that enables survival and production under saline and arid conditions [22,23]. Most of the feed consumed by camels in arid areas contains high levels of salt, which may affect the mineral absorption in the small intestine into the bloodstream. Elevated concentrations of Na^+^, Cl^−^, and other cations such as Ca^2+^ may alter blood pH, potentially impairing mineral absorption [24]. Therefore, the possibility of developing a supplementation program to avoid mineral deficiencies may be crucial in arid areas or areas where camels consume high salt and Ca^2+^ [24].

Scientific reports have shown that sufficient salt intake is essential for livestock health, particularly in hot arid regions where natural sodium sources are limited and water loss is frequent [25]. The relatively high salt requirements of camels are likely related to their adaptation to consuming desert plants and withstanding heat and water scarcity [26]. Despite the high salt level used in this study, which is estimated at 4.55%, about 29 g per day, this academic information is not translated into clear practical indications of the quantity, frequency, and form of salt to be given to modern camels.

It should be noted that the addition of high levels of salt to camels’ feed did not affect feed consumption, contrary to the results for young bulls, where the rate of concentrate intake in young bulls was reduced by 26% when high levels of 10% salt were added [27]. Scientific literature has shown that the intake of salt (NaCl) is crucial for the health of cattle, especially in hot arid areas with low sources of Na and frequent water losses [11]. The high salt demand in camel diets may be linked to the physiological mechanisms of camels, desert herbivores specialized in heat and water resistance [26,28].

The macro mineral Mg, K, Ca, Na, and P values in camel milk ranged within the reference interval in all physiological periods [21]. In the current study, camels fed a high-salt diet exhibited elevated serum Na, K, and P, accompanied by significant reductions in Ca and Mg. Macro mineral concentrations in milk showed similar patterns, although the inverse trends for Mg and Ca between serum and milk suggest that redistribution mechanisms operate to maintain milk composition under conditions of high dietary salinity. This may be related to the purebred nature of the animals, which have lower serum levels of Ca, Mg, and Na [24]. These findings highlight the camel’s capacity to adjust mineral partitioning between blood and milk as part of its adaptive strategy.

According to Goff [24], Ca^2+^, Mg^2+^, and phosphate (PO_4_^−3^) are the most common ions. In general, they are less effective in dietary absorption than Na^+^, K^+^, Cl^−^ and SO_4_^−2^, but are often present in relatively high quantities in feed. They may also contribute to ionic variations that influence blood pH. In theory, trace cations and anions from feed absorbed into the blood also affect the acid–base balance. However, their impact is negligible compared to the macro-minerals, and their presence is so marginal that it is not noticeable.

Correlation analyses further supported these observations. Phosphorus intake was negatively associated with milk Ca, whereas K intake showed a positive association with milk Ca. Milk Ca was negatively correlated with serum Mg but positively associated with milk Mg. Additional positive associations were also observed between milk Na and serum K, and between serum P and serum Na. These relationships confirm the strong interdependence among macro-minerals in camels, particularly between Ca–Mg and Na–K. Principal component analysis (PCA) reinforced these findings by demonstrating distinct clustering of minerals according to dietary treatment, reflecting coordinated regulatory mechanisms. Environmental factors may also have influenced mineral balance in camels. During summer, high ambient temperatures can increase Mg losses through sweating, thereby amplifying dietary effects. Similar impacts of heat stress on mineral balance have been reported in ruminants [29].

Ca is absorbed mainly by facilitated diffusion across the mucosa of the small intestine, a process mediated by calcium-binding proteins (CaBP). Although nutritionists traditionally consider Ca and P to be inextricably linked, this association arises primarily after absorption during bone metabolism; their respective intestinal absorption mechanisms are largely independent. P is absorbed mainly by passive diffusion along the electrochemical gradient in the small intestine, and this process is not strongly regulated hormonally.

Urea levels in the blood increased in camels that consumed high levels of salt, while triglycerides decreased as mediated by a period factor. The analysis of blood components may provide valuable information on the general health of the animal and serve as an indirect indicator of physiological status. Deviations in certain blood parameters from their normal ranges may provide clues for disease diagnosis [30].

In this study, the values of blood components were within the normal ranges reported for camels, including glucose concentrations of 60 to 140 mg/dL, total protein of 6.3 and 8.3 mg/dL, urea concentrations of 5 to 40 mg/dL, serum albumin concentrations of 25 to 45 g/L, globulin concentrations of 20 to 50 g/L [21], and triglyceride concentrations of 0.41–0.55 g/L [31]. Urea serves as an indirect test for kidney function and elimination [12]. Our results showed that urea levels in camels fed a high-salt diet were increased compared to the control. The elevated blood urea concentration in camels may be related to high salt intake, which increases renal filtration activity. Based on results for mineral content in milk, the percentages of mineral excretion in milk compared to intake were as follows: Mg (CON = 3.46% and T1 = 4.78%), K (CON = 39.09% and T1 = 45.74%), Ca (CON = 35.98% and T1 = 43.54%), Na (CON = 29.04% and T1 = 25.84%), and P (CON = 23.02% and T1 = 25.85%). Unfortunately, researchers were not able to take urine samples from individual animals to estimate the actual amount of minerals lost in the urine because of the difficulty in handling the camels.

During dehydration induced by high salt intake, the kidney actively participates in water regulation through the renin–angiotensin–aldosterone system (RAAS) and contributes to calcium and phosphorus metabolism via the formation of 1.25(OH)_2_-vitamin D_3_ and the regulation of acid–base balance [32]. Jaber et al. [33] stated that water restriction has a direct effect on the concentration of urea in the blood. They suggested that the lack of absorption increases the reabsorption of urea in the distal tubules and especially in the renal collecting ducts, and that the reabsorption of urea is therefore expected to increase as a highly permeable molecule. Moreover, insufficient water intake is expected to cause low blood pressure due to hypovolemia, leading to reduced renal blood flow, resulting in a reduced filtering rate and high urea concentration in the blood.

In addition, TG in blood serum can be modulated by energy levels in the diet, as reported by [34]. Furthermore, antioxidant stress may affect lipid profiles, as previously reported [31]. Jaber et al. [33] reported that triglycerides in sheep were significantly higher than those in camels. Feed restriction or malnutrition has been shown to markedly influence serum triglyceride levels due to changes in body fat mobilization. This massive lipolysis releases NEFA into the bloodstream and serves as an alternative source of energy for other tissues, maintaining the production of glucose. Water deprivation for four days has been shown to significantly increase blood triglyceride levels.

Overall, the findings demonstrate the complex regulation of mineral metabolism in camels under saline feeding conditions. They underscore the importance of establishing camel-specific mineral requirements and designing balanced supplementation strategies that address potential deficiencies, particularly of Ca and Mg. Such approaches will be essential for optimizing camel productivity, health, and reproductive performance in arid and semi-arid production systems. The limited number of studies on this topic poses challenges in interpreting the findings and comparing them with those reported for ruminants.

## 5. Conclusions

High dietary salt intake in lactating camels significantly altered mineral dynamics, with increased concentrations of K, Na, and P and significantly decreased Mg and Ca in serum. Conversely, the concentrations of Mg, K, and Ca were significantly higher in the milk of lactating camels that consumed high dietary salt. The contrasting directions of change between serum and milk highlight the presence of complex regulatory mechanisms governing mineral partitioning and homeostasis. Correlation and multivariate analyses further confirmed strong interactions among macro minerals, particularly between Ca and Mg and between Na and K. These findings demonstrate that camels exhibit unique mineral metabolism under saline feeding conditions, reflecting their remarkable adaptation to desert environments. In practice, the camel has been shown to prefer a high-salt diet and tolerate diets with high salt concentrations. Therefore, mineral supplementation strategies in camel diets should be carefully calibrated to prevent deficiencies, especially in the presence of high salt content.

## Figures and Tables

**Table 1:** Ingredients and chemical components (DM basis) for the two diets’ experimental including treatments CON with salt 1.5% and T1 with salt 4.5%.

Components %	Alfalfa Hay	CON	T1
Corn %	--	75	75
Barley %	--	15.6	12.6
soybean meal %	--	2.5	2.5
Molasses %	--	1.8	1.8
CMV %	--	0.9	0.9
Salt %	--	1.5	4.5
Ca carbonate %	--	2.7	2.7
**Chemical analysis**			
Dry matter(g/100 g)	89.29	91.29	90.96
Crude protein	13.2	10.6	10.2
Ether extract	1.03	2. 61	2.59
NDF	33.1	39.8	40.6
ADF	13.9	17.3	16.6
Ash	7.33	3.58	4.02
**Elements (µg/g)**			
K	1649.2	1591.2	1485.6
Na	2017.1	1511.29	1761.9
P	82.40	89.70	90.98
Mg	664.3	2416.4	2271.1
Ca	1960.1	2374.4	2334.6
DCAD ^1^ mEq/kg of DM	366.1	309.3	336.4

NDF: Neutral detergent fiber; ADF: Acid detergent fiber; K: Potassium; Na: Sodium; P: Phosphorus; Mg: Magnesium; Ca: Calcium; CMV: Complete minerals and vitamins premix. ^1^ The dietary cation-anion difference (DCAD) of a feed component was determined by DCAD (mEq) of (Na^+1^ + K^+1^) − (Cl^−1^ + SO_4_^−2^)/kg of DM [15].

**Table 2:** Milk production at a.m. and p.m., and water consumed in camels fed two dietary treatments: CON with salt 1.5% and T1 with salt 4.5% during two periods.

Element’s µg/mL	Treatment		SEM	*p* Value		
	CON-Salt 1.5%	T1-Salt 4%		Treat	Period	T × P
Milk yield (AM)	1.88	1.92	0.16	0.86	0.29	0.78
Milk yield (PM)	2.16	2.62	0.18	0.09	0.17	0.33
Water intake (L/day)	31.88 ^b^	34.01 ^a^	0.10	0.01	0.01	0.03

^a,b^: the mean values for the different letters vary significantly between milk yield (AM and PM) and water intake in two treatments fed by salt 1.5 and 4.5% supplement (*p* ≤ 0.05). SEM: standard error of mean.

**Table 3:** Concentrations of macro-mineral in camels’ serum (µg/mL) fed two dietary treatments CON with salt 1.5% and T1 with salt 4.5%during two periods as a crossover trial.

Element’s µg/mL	Treatment		SEM	*p* Value		
	CON-Salt 1.5%	T1-Salt 4.5%		Treat	Period	T × P
Mg	156.69 ^a^	123.89 ^b^	7.443	0.01	0.55	0.77
K	274.67	307.51	13.71	0.25	0.50	0.07
Ca	1032.30 ^a^	884.37 ^b^	73.57	0.05	0.38	0.74
Na	253.55	291.70	25.70	0.44	0.79	0.10
P	6.46	7.35	0.742	0.19	0.76	0.74

^a,b^: the mean values for the different letters vary significantly between macro-minerals in two treatments feed by salt 1.5 and 4.5% supplement (*p* ≤ 0.05). SEM: standard error of the mean.

**Table 4:** Concentrations of macro-mineral in camels’ milk (µg/mL) fed two dietary treatments CON with salt 1.5% and T1 with salt 4.5% during two period.

Element’s µg/mL	Treatment		SEM	*p* Value		
	CON-Salt 1.5%	T1-Salt 4%		Treat	Period	T × P
Mg	106.68 ^b^	140.36 ^a^	9.33	0.02	0.99	0.68
K	1266.75 ^b^	1433.78 ^a^	56.50	0.03	0.89	0.63
Ca	1559.73 ^b^	1870.03 ^a^	81.45	0.01	0.53	0.79
Na	1024.71	976.52	36.20	0.38	0.23	0.37
P	39.62	44.83	2.41	0.21	0.25	0.57

^a,b^: the mean values for the different letters vary significantly between macro-minerals in two treatments fed by salt 1.5 and 4.5% supplements (*p* ≤ 0.05). SEM: standard error of mean.

**Table 5:** Blood metabolites (mg/dL) in camels’ milk fed two dietary treatments: CON with salt 1.5% and T1 with salt 4.5% during two periods.

Item	Treatment		SEM	*p* Value		
	CON-Salt 1.5%	T1-Salt 4.5%		Treat	Period	T × P
GLC mg/dL	51.27	50.81	3.44	0.92	0.99	0.38
TP mg/dL	43.32	43.63	5.43	0.96	0.57	0.06
ALB mg/dL	3.86	3.67	0.34	0.71	0.40	0.92
GLB mg/dL	39.46	39.96	5.38	0.94	0.70	0.06
A/G	0.12	0.11	0.02	0.72	0.83	0.08
Urea mg/dL	41.03 ^b^	42.94 ^a^	4.41	0.76	0.004	0.15
TG mg/dL	34.11 ^b^	27.24 ^a^	2.43	0.05	0.03	0.83

^a,b^: the mean values for the different letters vary significantly between blood metabolites in two periods (*p* ≤ 0.05). SEM: standard error of mean. GLC: glucose; TP: total protein; ALB: albumin; GLB: globulin; TG: triglycerides.

## Data Availability

The original contributions presented in this study are included in the article. Further inquiries can be directed to the corresponding author.
